# Outcome of adalimumab monotherapy in paediatric non-infectious uveitis

**DOI:** 10.1186/s12969-023-00794-y

**Published:** 2023-03-02

**Authors:** DA Al-Julandani, NK Bagri, N Tsang, S Clarke, A Upadhyay, C Guly, AV Ramanan

**Affiliations:** 1 Department of Paediatric Rheumatology, Bristol Royal Hospital for Children, Upper Maudlin Street, Bristol, UK; 2 Division of Paediatric Rheumatology, Department of Paediatrics, All India Institute of Medical Sciences, New Delhi, India; 3 University of Bristol, Bristol, UK; 4 School of Population Health Sciences, University of Bristol, Bristol, UK; 5 MRC Integrative Epidemiology Unit, University of Bristol, Bristol, UK; 6 Scientist II CRU, All India Institute of Medical Sciences, New Delhi, India; 7 Bristol Eye Hospital, University Hospitals Bristol and Weston NHS Foundation Trust, Bristol, UK; 8 Translational Health Science, University of Bristol, Bristol, UK

**Keywords:** Non-infectious uveitis, Paediatrics, Adalimumab monotherapy, JIA-uveitis

## Abstract

**Background:**

Adalimumab in combination with other disease-modifying antirheumatic drugs (DMARD) such as methotrexate has a proven efficacy in the management of paediatric non-infectious uveitis. However, many children experience significant intolerance to methotrexate while on this combination, leaving a dilemma for clinicians for choosing the subsequent therapeutic roadmap. Continuation of adalimumab monotherapy might be an alternative feasible option under such settings. This study aims to investigate the efficacy of adalimumab monotherapy in paediatric non-infectious uveitis.

**Methods:**

Children with non-infectious uveitis on adalimumab monotherapy (from August 2015 to June 2022) following intolerance to accompanying methotrexate or mycophenolate mofetil were included in this retrospective study. Data were collected at the initiation of adalimumab monotherapy and at three monthly intervals until the last visit. The primary outcome was to evaluate disease control on adalimumab monotherapy as determined by the proportion of patients who had less than a 2-step worsening in uveitis (as per SUN score) and no additional systemic immunosuppression during follow-up. Secondary outcome measures were visual outcome, complications and side-effect profile of adalimumab monotherapy.

**Results:**

Data was collected for 28 patients (56 eyes). The most common uveitis type and course were anterior and chronic uveitis respectively. Juvenile idiopathic arthritis-associated uveitis was the most common underlying diagnosis. During the study period, 23 (82.14%) of the study subjects met the primary outcome. On Kaplan–Meier survival analysis 81.25% (95% CI; 60.6–91.7%) children maintained remission at 12 months on adalimumab monotherapy.

**Conclusion:**

Continuation of adalimumab monotherapy is an effective therapeutic option for the treatment of non-infectious uveitis in children who are intolerant to the combination of adalimumab and methotrexate or mycophenolate mofetil.

## Key messages

**What is already known on this topic:** Adalimumab in combination with methotrexate is effective in the treatment of non-infectious uveitis. However

There is limited evidence on adalimumab monotherapy for the management of uveitis.

**What this study adds:** This retrospective study demonstrates the efficacy of adalimumab monotherapy in children with non-infectious uveitis who are intolerant to other immunosuppressants, particularly methotrexate at medium-term follow-up.

## Introduction

Paediatric uveitis has an incidence per /100000 population/year of 4.3 and a prevalence of 27.9 [1]. Whilst Juvenile idiopathic arthritis (JIA) accounts for approximately 41–47% of paediatric uveitis, 28–51% remains idiopathic [2]. Uncontrolled uveitis can lead to various sight-threatening complications such as vitreous haze, posterior synechiae, cataract, band keratopathy, macular oedema and glaucoma [3].

Methotrexate has been the most frequently used disease-modifying antirheumatic drug (DMARD) for paediatric uveitis with an overall probability of 73% in improving intraocular inflammation [4, 5]. Though widely used for uveitis, methotrexate fails to control inflammation in nearly 40% of children [4]. Two pivotal randomised controlled trials SYCAMORE and ADJUVITE have demonstrated the promising efficacy of the addition of Adalimumab to methotrexate in uveitis and presently addition of adalimumab is the standard of care for the management of uveitis refractory to first-line DMARDs like methotrexate. Although effective, this combination is often challenged by intolerance or side effects related to  methotrexate [5, 6]. Continuation of adalimumab monotherapy may be a potential therapeutic option in this setting, however, there is paucity of data in this regard. In this retrospective study we aim to investigate the effectiveness of adalimumab monotherapy in children with non-infectious uveitis who discontinued methotrexate or other immunosuppressants.

## Methods

### Patient identification

For this retrospective study, de-identified data of children aged less than 16 years with non-infectious uveitis on adalimumab monotherapy visiting the Paediatric Rheumatology-uveitis clinic, at Bristol University hospital from August 2015 to June 2022, were collected in a predesigned proforma. This study was provided ethical waiver as per Institute norms.

### Data collection

Data were collected at regular time intervals; at baseline i.e. initiation of adalimumab monotherapy and subsequently at 3 monthly until the last visit. Patient’s demographic details including gender, ethnicity, underlying diagnosis, age at diagnosis, type, course and laterality of uveitis and laboratory investigations were recorded. Pharmacological treatment prior to adalimumab monotherapy and reasons for cessation of concomitant medications were noted. The number of steroid drops used at baseline and subsequent follow-ups were recorded. Uveitis activity was defined by the Standardisation of Uveitis Nomenclature (SUN)-Working Group Activity. Inactive uveitis was defined as < 1 cell per field on standard slit-lamp examination [7]. Visual outcome was measured by the number of cells, presence of flare, visual acuity (as per logMAR), binocular indirect ophthalmoscopy (BIO) score [7, 8], intraocular pressure (IOP), and posterior segment inflammation. Elevated IOP was defined as > 21 mmHg. Visual impairment is graded into logMAR ≤ 0.3, > 0.3 logMAR and > 1.0 logMAR. Ocular complications and surgery were noted at baseline and during monotherapy. The primary outcome was to evaluate disease control on adalimumab monotherapy which was assessed by the proportion of patients who had less than a 2-step worsening in anterior uveitis (as per SUN score) or BIO score (for intermediate uveitis) with no addition of systemic immunosuppression during follow-up. Secondary outcome measures were visual outcome, complications and side-effect profile. Treatment failure was defined as the need for additional systemic immunosuppressant medication for active uveitis during follow-up.

### Statistical analysis

De-identified data was entered in an excel sheet. Statistical analyses were performed using R (version 4.0.2) in R Studio (1.3.1073). Patient demographics and clinical characteristics are presented using descriptive statistics. For continuous variables, density plots were visually inspected, and the data was presented as mean (standard deviation, SD) for normally distributed data, or median (interquartile range, IQR) where data were skewed. Responders and non-responders to adalimumab monotherapy were compared using Fisher’s exact test for categorical variables and Welch’s t-test for continuous variables. Kaplan -Meir survival analysis was plotted for estimating the proportion of children meeting the primary outcome.

## Results

Twenty-eight patients (56 eyes) were enrolled during the study period. Table 1 represents the baseline characteristics of the study population. All patients received methotrexate before adalimumab monotherapy, while 19 were exposed to adalimumab prior to monotherapy. Methotrexate was discontinued due to intolerance (frequent nausea, vomiting) and transaminitis in 26 and 2 subjects respectively. Three children also received mycophenolate mofetil (MMF) which was discontinued due to diarrhoea (*n* = 1), intolerance/fatigue (*n* = 1) and miscommunication (*n* = 1). One child with JIA received etanercept with methotrexate for control of her arthritis but switched to adalimumab when she developed uveitis.

**Table 1:** Baseline characteristics of the study population (*n* = 28)

Characteristic	*n* (%)
Gender, Female, *n* (%)	15 (53.6)
Ethnicity, *n* (%)	
White-British	24 (85.7)
Others	4 (14.3)
Age at diagnosis of uveitis, years median,(IQR)	5.6 (6.5)
Anatomical distribution of uveitis, *n* (%)	
Anterior	24 (82.8)*
Intermediate	5 (17.2)*
Presentation of uveitis, *n* (%)	
Symptomatic	3 (10.7)
Asymptomatic	25 (89.3)
Laterality, *n* (%)	
Bilateral	21 (75.0)
Unilateral	7 (25.0)
Underlying diagnosis, *n* (%)	
JIA	15 (53.6)
Idiopathic	13 (46.4)
ANA positivity, *n*/*N* ( %)	9/11 (99%)
Duration of total adalimumab therapy (months), median (IQR)	34.5 (41.25)
Duration of adalimumab monotherapy (months), median, (IQR)	15 (18.75)

^*^One child had anterior and intermediate uveitis

**Fig. 1:**
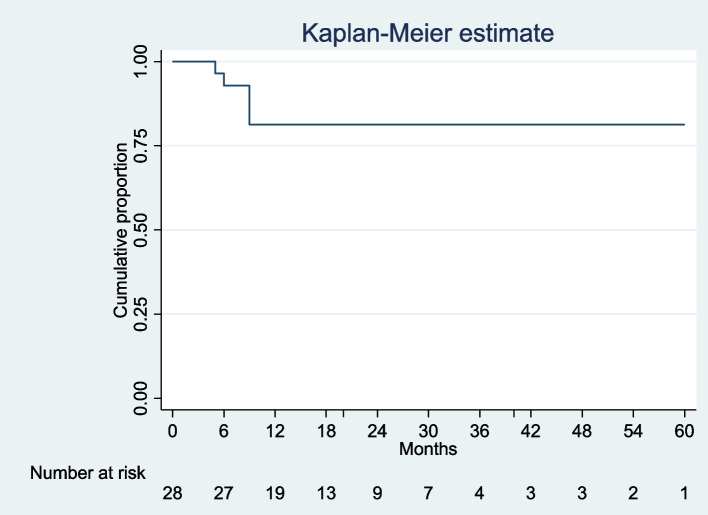
Kaplan–Meier survival estimate for proportion of children maintaining control of uveitis on adalimumab monotherapy

Of the 28 patients who received adalimumab monotherapy, 23 (82.14%) met the primary outcome i.e., they had less than a two-step worsening in uveitis necessitating the addition of another systemic immunosuppressant, while 5(17.86%) failed adalimumab monotherapy and required the addition of another systemic immunosuppressant. On Kaplan–Meier survival analysis 81.25% (95% CI; 60.6–91.7%) children maintained remission at 12 months of on adalimumab monotherapy (Fig. 1).

Among responders, adalimumab was discontinued in nine subjects (9/23) after 20(19.5) [median (IQR)] months of sustained remission. 2/9 patients flared within 6 months of stopping adalimumab and the adalimumab was reintroduced. In the remaining 14 subjects, the uveitis was controlled until last follow-up at 19 (21) [median (IQR)] months of adalimumab monotherapy. Nine patients (14 eyes) had AC cells at baseline; 9 eyes; 0.5 + , 4 eyes; 1 + ; and one eye had 3 + cells. Twelve eyes were on steroid drops (mean 3, range 1–6). All these 14 eyes become inactive at 6 (3.5) [median (IQR)] months’ follow-up. Twelve patients (12/23) (15eyes) had mild flares (0.5 +—1 +) during the period of adalimumab monotherapy which were either observed (*n* = 10 eyes) or managed with short course of steroid drops (*n* = 5 eyes).

At the last follow-up, only 2 eyes (in 2 patients) required topical steroids; one eye was inactive on a tapering schedule of one drop/day while the other one was active (1 +) requiring 4 drops/day. Visual acuity was maintained at last follow up ≤ 0.3 Log MAR in all except one eye which had a worsening from 0.2 to 0.7 on Log MAR. None of the eyes had increased IOP. Among the 23 children (46 eyes) who responded, 13 eyes had one or more ocular complications at baseline; cataract (*n* = 7), amblyopia (*n* = 5), glaucoma (*n* = 3), posterior synechiae (*n* = 2), band keratopathy (*n* = 1) and retinal detachment (*n* = 1). No new complications were observed during the follow-up.

Five patients failed adalimumab monotherapy. The median (IQR) time to failure was 9 (3.5) months from adalimumab monotherapy. MMF and methotrexate were added in 4 and 1 patient respectively; the patient on methotrexate was later shifted to MMF due to methotrexate intolerance. One patient on MMF was later switched to tocilizumab due to arthritis and the development of adalimumab antibodies. The anti-drug antibodies were not available for other subjects.

Comparisons between responders and non-responders to adalimumab monotherapy were undertaken (Table 2). More children with anterior uveitis compared to intermediate uveitis responded to adalimumab monotherapy. There were no other significant predictors of response to adalimumab monotherapy. No adverse event related to adalimumab was noted during the study period.

**Table 2:** Predictors of response

	Failed adalimumab monotherapy (*N* = 5)	Maintained adalimumab monotherapy (*N* = 23)	*p* value
Gender, Female, *n* (%)	3 (60.0)	12 (52.2)	1.00
Ethnicity			
White British, *n* (%)	3 (60.0)	21 (91.3)	0.14
Others, *n* (%)	2 (40.0)	2 (8.7)	
Anatomical distribution of uveitis type, *n* (%)			
Anterior	3 (60.0)*	21 (91.3)	< 0.05
Intermediate	3 (40.0)*	2 (8.7)	
Uveitis presentation, *n* (%)			
Symptomatic	0 (0)	3 (13.0)	1.00
Asymptomatic	5 (100)	20 (87.0)	
Uveitis laterality, *n* (%)			
Unilateral	2 (40.0)	5 (21.7)	0.57
Bilateral	3 (60.0)	18 (78.2)	
Underlying diagnosis, *n* (%)			
JIA	2 (40.0)	13 (56.5)	0.64
Idiopathic	3 (60.0)	10 (43.5)	
ANA positive JIA patients (*n*/*N*)	2/2	5/5	1.00
Age at diagnosis of uveitis (years), median (IQR)	5.0 (7.0)	6.0 (6.0)	0.89
Uveitis duration before adalimumab monotherapy (months), median (IQR)	50.0 (38.0)	48.0 (34.5)	0.68
Total duration of adalimumab (including monotherapy period, months) median (IQR)	39.0 (34.0)	33.0 (41.0)	0.71
Duration of adalimumab before monotherapy, months, median (IQR)	30 (30)	17 (28)	0.10
Duration of adalimumab monotherapy, months, median (IQR)	9 (3.5)	20 (19)	< 0.05
Type of Immunosuppressive drug prior to adalimumab monotherapy, n (%)			
MTX	5 (100)	16 (70.0)	0.29
MMF	0 (0)	0 (0)	1.00
MTX plus MMF	0 (0)	6 (26.1)	0.55
Systemic steroid	0 (0)	0 (0)	1.00
Etanercept	0 (0)	1 (4.3)	1.00
Baseline AC cells (number of patients/ number of eyes)			
0	5/10	32/46	0.28^#^
0.5 +	5/10	9/46	
1 +	0/10	4/46	
2 +	0/10	0/46	
3 +	0/10	1/46	
4 +	0/10	0/46	
Baseline Intraocular pressure, *n* (%)			
Normal	9 (90.0)	46 (100)	0.18
> 21 mmHg	1 (10.0)	0 (0)	
Baseline Number of glucocorticoid drops per eye per day, n (%)			
≤ 3	10 (100)	40 (87.0)	0.58
> 3	0 (0)	6 (13.0)	
Baseline Visual Impairment (number of eyes)			
≤ 0.3 logMAR	10/10	46/46	1.00
> 0.3 logMAR	0/10	0/46	
Baseline Ocular complication/surgeries (number of patients)	4/5	8/23	1.00
Cataract	2/5	4/23	0.29
Band keratopathy	1/5	0/23	0.18
Retinal detachment	0/5	1/23	1.00
Glaucoma	1/5	1/23	1.00
Posterior synechiae	0/5	2/23	1.00
Ocular procedure/surgery	1/5	3/23	1.00

^*^One child had anterior and intermediate uveitis

^#^*P* value represents patients with flare (defined as > / = 0.5 + AC cells) versus no flare (0 AC cells) at baseline in the two groups

## Discussion

This retrospective study demonstrates the efficacy of adalimumab monotherapy in paediatric uveitis. Eighty-two % of patients, on adalimumab monotherapy following intolerance to methotrexate or mycophenolate did not require an additional systemic immunosuppressant.

Adalimumab in combination with methotrexate is effective in treating non-infectious uveitis. The recent ACR and SHARE guidance for management of JIA associated uveitis recommends addition of anti-TNF for control of JIA-associated uveitis, which is refractory to methotrexate [9, 10]. In a pivotal randomised controlled trial, Ramanan et al. [11] has demonstrated that Adalimumab therapy in combination with stable doses of methotrexate-controlled inflammation and was associated with a lower rate of treatment failure than placebo (27% vs 60% respectively) among children with active JIA-associated uveitis. In this study, the adalimumab group showed a significantly longer mean time of sustained inactive uveitis (0 cells) than those in placebo group.

Although effective, methotrexate has a myriad of adverse-effects (particularly gastrointestinal) leading to intolerance. Various studies have shown that nearly 40–50% of children with JIA suffer gastrointestinal and behavioural side-effects with methotrexate [12, 13] thus posing a challenge in continuing it. In our study the patients who stopped methotrexate did so due to intolerance (92.86%) or transaminitis (7%).

Studies on adalimumab monotherapy for management of non-infectious uveitis are limited. In a retrospective multicentre study by Bitossi et al. [14] assessing the long-term ocular control of adalimumab alone or in combination of DMARD in non-infectious uveitis found that 94.6% of patients had satisfactory ocular control and the concomitant use of DMARDs does not provide additional benefits to adalimumab monotherapy in terms of control of ocular inflammation, steroid sparing, or drug retention rates. Adalimumab monotherapy has been tried in other inflammatory conditions such as psoriatic arthritis and Crohn’s disease with conflicting results. Matsumoto et al. [15] showed that the efficacy of a combination of adalimumab and azathioprine did not vary from that of adalimumab monotherapy in patients with Crohn's disease who had never received either drug. In contrast, the combination therapy of adalimumab and methotrexate displayed a trend toward a better PASI 75 response and significantly lower adalimumab anti-drug antibodies than adalimumab monotherapy in psoriasis [16].

Similar to Bitossi et al. [14] study which demonstrated the favourable outcomes with adalimumab in adults with non-infectious uveitis, we also observed 82.14% of children achieved uveitis control on adalimumab monotherapy without any new complications or side effects. All eyes which were active at baseline became inactive during the 3–9 months follow-up and only 2 eyes required prednisolone drops at the last follow-up.

One potential benefit of additional immunosuppression with adalimumab is the potential to reduce the development of drug antibodies [17, 18]. Although our study's results of adalimumab monotherapy are promising, the long-term efficacy of adalimumab monotherapy and the rate of adalimumab antibody development on adalimumab monotherapy is unknown.

Limitations of the study include the retrospective design and a small sample size. The adalimumab drug levels and antibody levels to adalimumab after initiation of adalimumab monotherapy were not measured in this study.

In conclusion, this study has demonstrated that adalimumab monotherapy is a safe and effective treatment for paediatric non-infectious uveitis at the medium-term follow-up, and is a reasonable option for children who are intolerant to methotrexate or mycophenolate. Long-term data is required to determine whether adalimumab monotherapy provides persistent control of uveitis. The role of adalimumab as a primary treatment in childhood uveitis also deserves further study.

## Data Availability

Data access is subject to Institutional processes.
